# Field evaluation of four widely used mosquito traps in Central Europe

**DOI:** 10.1186/1756-3305-7-268

**Published:** 2014-06-12

**Authors:** Renke Lühken, Wolf Peter Pfitzner, Jessica Börstler, Rolf Garms, Katrin Huber, Nino Schork, Sonja Steinke, Ellen Kiel, Norbert Becker, Egbert Tannich, Andreas Krüger

**Affiliations:** 1 Department of Molecular Parasitology, Bernhard Nocht Institute for Tropical Medicine, Hamburg, Germany; 2 Research Group Aquatic Ecology and Nature Conservation, Carl von Ossietzky University, Oldenburg, Germany; 3 German Mosquito Control Association (KABS e.V.), Institute for Dipterology, Waldsee, Germany; 4 Department of Virology, Bernhard Nocht Institute for Tropical Medicine, Hamburg, Germany; 5 University of Heidelberg, Heidelberg, Germany; 6 German Centre for Infection Research, partner site Hamburg-Luebeck-Borstel, Hamburg, Germany; 7 Department of Tropical Medicine, Bundeswehr Hospital Hamburg, Hamburg, Germany

## Abstract

**Background:**

To monitor adult mosquitoes several trapping devices are available. These are differently constructed and use various mechanisms for mosquito attraction, thus resulting in different trapping sensitivities and efficacies for the various species. Mosquito monitoring and surveillance programs in Europe use various types of mosquito traps, but only a few comparisons have been conducted so far. This study compared the performance of four commercial trapping devices, which are commonly used in Europe.

**Methods:**

Four different traps, Biogents Sentinel trap (BG trap), Heavy Duty Encephalitis Vector Survey trap (EVS trap), Centres for Disease Control miniature light trap (CDC trap) and Mosquito Magnet Patriot Mosquito trap (MM trap) were compared in a 4 × 4 latin square study. In the years 2012 and 2013, more than seventy 24-hour trap comparisons were conducted at ten different locations in northern and southern Germany, representing urban, forest and floodplain biotopes.

**Results:**

Per 24-hour trapping period, the BG trap caught the widest range of mosquito species, the highest number of individuals of the genus *Culex* as well as the highest number of individuals of the species *Ochlerotatus cantans*, *Aedes cinereus*/*geminus*, *Oc. communis* and *Culex pipiens*/*torrentium*. The CDC trap revealed best performance for *Aedes vexans,* whereas the MM trap was most efficient for mosquitoes of the genus *Anopheles* and the species *Oc. geniculatus*. The EVS trap did not catch more individuals of any genus or species compared to the other three trapping devices. The BG trap caught the highest number of individuals per trapping period in urban environments as well as in wet forest, while the CDC trap caught the highest number of individuals in the floodplain biotopes. Additionally, the BG trap was most efficient for the number of mosquito species in urban locations.

**Conclusion:**

The BG trap showed a significantly better or similar performance compared to the CDC, EVS or MM trap with regard to trapping efficacy for most common mosquito species in Germany, including diversity of mosquito species and number of mosquitoes per trapping period. Thus, the BG trap is probably the best solution for general monitoring or surveillance programs of adult mosquitoes in Central Europe.

## Background

Most mosquito monitoring and surveillance programs include the monitoring of adults using different types of trapping devices. Due to automatic trapping by aspiration, mosquito traps have the advantage of relative low costs for data collection in combination with a constant effort independent of the operator, resulting in comparable samples from different trapping sites. Therefore, adult traps are commonly used for the inventory of mosquito biodiversity [1], surveillance of invasive mosquitoes at potential introduction sites [2], monitoring of mosquito-borne pathogens [3], or the reduction of mosquito nuisance [4]. However, in the course of increasing attention for mosquitoes due to the worldwide spread of invasive mosquitoes [5-7] and mosquito-borne pathogens [8], also the number of commercially available traps increased, which are distributed as tools for scientific studies or for mosquito control [4,9,10]. These trapping devices use various cues for mosquito attraction (e.g. carbon dioxide, heat, water vapour, olfactory lures, or visual cues), which may influence trapping efficacies for the different genera or species [11].

Previous studies on the comparison of mosquito traps were predominantly conducted in North and South America [12-14]. Many of these studies focused primarily on the effectiveness of the traps to catch invasive and/or highly vector-competent species (e.g. *Aedes albopictus*) [14,15]. Due to the spread of invasive mosquitoes [16] and mosquito-borne pathogens (e.g. West Nile virus [17]) in Europe, mosquito monitoring activities have substantially increased during recent years [2,18], but only a few studies have compared the efficacy of different mosquito traps for this region. The Mosquito Magnet Commercial Pro caught more mosquito individuals and a wider range of species than the Centres for Disease Control miniature light trap (CDC trap) in Great Britain [19]. In contrast, Reusken *et al.*[20] found that the CDC trap performed better than the Mosquito Magnet Liberty in the Netherlands. A limited study in Germany compared the Bidirectional Fay-Prince Trap, Biogents Sentinel (BG trap) and Mosquito Magnet Liberty, but did not find significant differences [21]. The most comprehensive comparison of mosquito traps was conducted in northern Italy with the experimental Biogents BG Eisenhans de Luxe, CDC trap and two mosquito traps for the reduction of mosquito nuisance (Acti Power Trap PV 440 and Acti Power Trap MT 250 Plus) [10]. For the collection of *Aedes albopictus*, a better trapping efficacy was found for the Biogents BG Eisenhans de Luxe compared to the other three trapping devices. Differences between the BG and CDC traps were reported only for *Anopheles atroparvus* during a trap comparison in Spanish wetlands [22].

Previous nationwide monitoring programs of mosquito species in Europe used different trapping devices, e.g. Mosquito Magnet Liberty Plus in Switzerland [1], the CDC trap and Mosquito Magnet counter-flow trap in Sweden [23], Mosquito Magnet Liberty Plus in Belgium, Netherlands and Luxembourg [1,24], or Heavy Duty Encephalitis Vector Survey trap and BG traps in Germany [25]. However, a comprehensive comparison of trapping efficacies of these adult mosquito traps commonly used in Central Europe has not been conducted and the choice between the different trapping devices is based on expert judgment or studies from other regions [1]. Therefore, the present study aimed to compare four trapping devices for mosquito adults. Our objectives were (i) to compare the efficacies of traps concerning the variety of mosquito species and the overall number of mosquitoes, as well as (ii) to identify the most efficient trap for different biotopes.

## Methods

**Figure 1:**
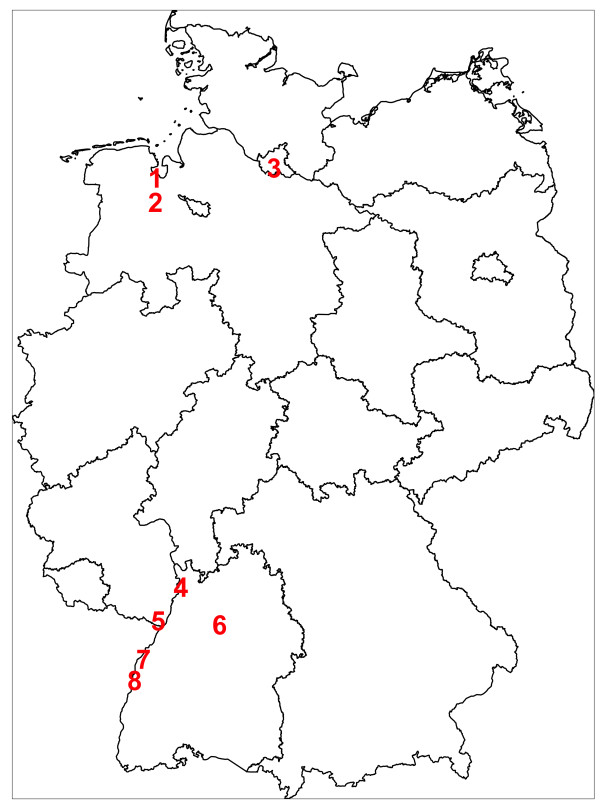
**Sampling locations.** Sampling locations of the trap comparisons in Germany. Numbers correspond to the IDs in Table 1.

Trap comparisons were conducted in the years 2012 and 2013 during 19 sampling periods in ten different locations in northern (3 locations) and southern Germany (7 locations) (Table 1, Figure 1). Locations in northern Germany included gardens in urban areas and a cattle farm, and in southern Germany floodplain areas, a wet forest, a cemetery in an urban environment, and the edge of a wood in an urban environment.

**Table 1:** Sampling locations

**ID**	**Description**	**Aggregated biotop**	**Sampling period**	**Temperature during sampling period [°C] (mean, minimum-maximum range)**	**Precipitation during sampling period [mm] (mean, minimum-maximum range)**
1	Garden in an urban area	Urban	05.09.-09.09.2012	16.9 (8.4-28.4)	0.0 (0.0-0.0)
			10.06.-14.06.2013	15.4 (5.1-24.3)	2.1 (0.0-8.5)
			30.07.-03.08.2013	22.2 (13.9-34.9)	1.0 (0.0-4.6)
2	Cattle farm within a suburban environment	Urban	19.08.-23.08.2013	16.9 (9.2-24.1)	3.6 (0.0-18.0)
			26.08.-30.08.2013	17.3 (7.5-24.3)	0.0 (0.0-0.0)
3	Garden in an urban area	Urban	03.06.-06.06.2012	10.2 (4.9-15.1)	1.4 (0.0-3.9)
			09.07.-13.07.2012	16.0 (10.4-22.8)	5.2 (0.0-8.2)
			28.08.-01.09.2012	16.2 (8.8-24.9)	0.2 (0.0-0.6)
			10.06.-14.06.2013	15.6 (4.9-24.3)	3.8 (0.0-14.8)
			08.07.-12.07.2013	17.2 (10.3-25.4)	0.0 (0.0-0.0)
4	Forest in river inundation area	Floodplain	17.07.-21.07.2012	18.0 (10.0-27.2)	1.9 (0.0-5.3)
			24.07.-28.07.2012	22.6 (2.1-33.3)	6.5 (0.0-16.8)
			03.08.-07.08.2012	20.4 (11.4-32.3)	0.1 (0.0-0.7)
			13.08.-17.08.2012	21.2 (9.4-33.6)	1.9 (0.0-9.6)
5a	Mixed forest	Wet forest	04.07.-08.07.2013	20.8 (12.1-28.8)	0.1 (0.0-0.5)
5b	Mixed forest	Wet forest	04.07.-08.07.2013	20.8 (12.1-28.8)	0.1 (0.0-0.5)
6a	Cemetery within an urban environment	Urban	26.08.-30.08.2013	17.4 (9.2-26.1)	0.0 (0.0-0.0)
6b	Edge of a wood within an urban environment	Urban	26.08.-30.08.2013	17.4 (9.2-26.1)	0.0 (0.0-0.0)
7	Forest in river inundation area	Floodplain	19.08.-23.08.2013	23.6 (10.5-35.2)	0.3 (0.0-1.3)
			07.09.-11.09.2012	18.4 (5.4-30.3)	3.2 (0.0-15.9)
8	Forest in river inundation area	Floodplain	02.09.-06.09.2012	17.9 (7.6-26.7)	0.0 (0.0-0.0)

Characterisation of the sampling locations and sampling periods. The temperature and precipitation during the sampling period were derived from the nearest weather station [26].

Four different traps were compared, which all have been developed to collect host-seeking mosquitoes by aspiration, but differ in their mechanisms of attraction and trapping: (1) Biogents Sentinel trap (BG trap) (BioGents, Regensburg, Germany, http://www.biogents.com/) with BG Lure sachets (BioGents, GmbH, Regensburg, Germany, http://www.biogents.com/) and CO_2_ from a gas cylinder, (2) Heavy Duty Encephalitis Vector Survey trap (EVS trap) (BioQuip Products, Rancho Dominguez, California, USA; http://www.bioquip.com/) with CO_2_ from dry ice (2.5 kg per 24 hours) and without EVS trap lamp, (3) Centres for Disease Control miniature light trap (CDC trap) (BioQuip Products, Rancho Dominguez, California, USA; http://www.bioquip.com/) with CDC bulb and with CO_2_ from dry ice (2.5 kg per 24 hours), which was also put in EVS dry ice containers above the trap, and (4) the Mosquito Magnet Patriot Mosquito trap (MM trap) (MosquitoMagnet, Lititz, Pennsylvania, USA; http://www.mosquitomagnet.com/) with R-Octenol (MosquitoMagnet, Lititz, Pennsylvania, USA; http://www.mosquitomagnet.com/). The MM trap converts propane into CO_2_. EVS and CDC traps were hung on low trees or wooden posts (trap opening approximately at 1 m height), whereas the BG and MM traps were placed on the ground following manufacturers instructions.

A 4 × 4 latin square experimental design was applied. At each location, all traps were placed approximately 50 m from each other at four different sampling points. Every 24 hours, all traps were rotated to the next position to reduce sampling point specific differences. One complete trapping cycle per latin square consisted of four 24-hour trapping periods. Mosquitoes were collected every 24 hours in the late afternoon, killed in a freezer and morphologically identified in the laboratory [27,28]. Four morphologically very similar species were summarized as species pairs (*Aedes cinereus*/*geminus*, *Ochlerotatus excrucians*/*annulipes*, *Ochlerotatus sticticus*/*diantaeus* and *Culex pipiens*/*torrentium*), because a morphological differentiation is not possible or doubtful in cases where the material is in poor condition. In terms of the taxonomy of Aedini species, the generic names used here follow the system of Becker *et al.*[27,28] and are not adopted from the revisions of Reinert *et al.*[29].

Generalized linear mixed models (GLMM) were used to analyse the effect of different trapping devices on the number of caught individuals for all species/genera per trapping period, total number of individuals/species per trapping period and total number of caught individuals/species per trapping period differentiated for aggregated biotopes. GLMMs allow dependent variables to be modelled while controlling for independent random variables (in this case the latin square number) to test the statistical significance of a fixed independent variable (type of trapping device). Mean and standard errors of differences in least squares means associated with a mixed linear model were calculated. Furthermore, Simpson’s diversity index per trapping period was caculated to compare the recorded species diversity among the four trapping devices. Data preparation, visualization and statistical analyses were conducted with R [30] using functions from the packages ggplot2 [31], lm4 [32], lmerTest [33], plyr [34], sp [35,36], and vegan [37].

## Results

A total of 83 trap comparisons were conducted. However, due to organisational and technical issues, nine trapping periods comprised only three different trapping devices (BG trap, CDC trap, and EVS trap), thus resulting in 323 24-hour sampling periods (83 × BG trap, 83 × CDC trap, 83 × EVS trap, 74 × MM trap).

**Figure 2:**
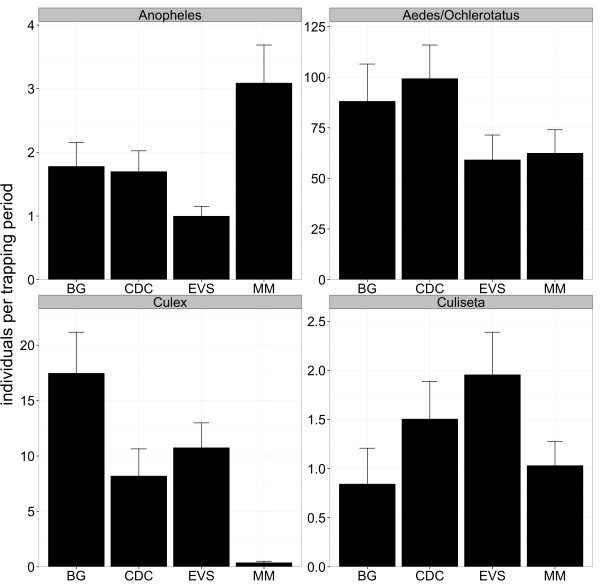
**Number of trapped individuals per genera among the four trapping devices.** Mean +/-SE number of trapped individuals per trapping period among the four trapping devices. Only mosquito genera caught with more than 100 individuals are shown and trapping periods were only included if the genus was detected with at least one individual in the corresponding trapping period at the sampling location.

**Figure 3:**
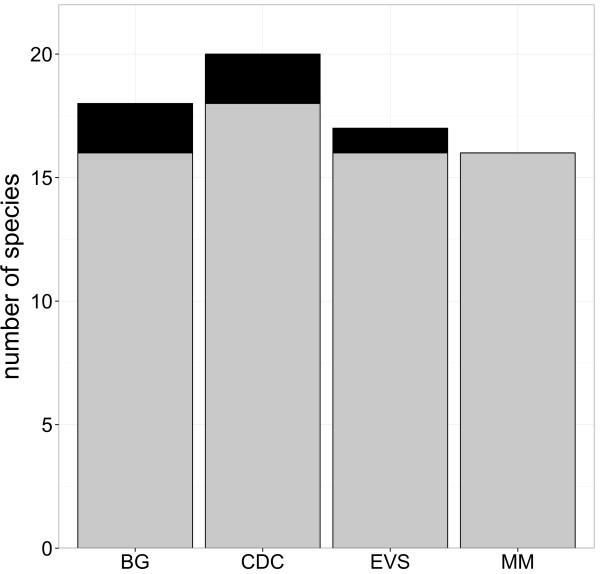
**Number of species among the four trapping devices.** Total number of species caught among the four trapping devices (grey = number of species without singletons, black = singletons).

**Figure 4:**
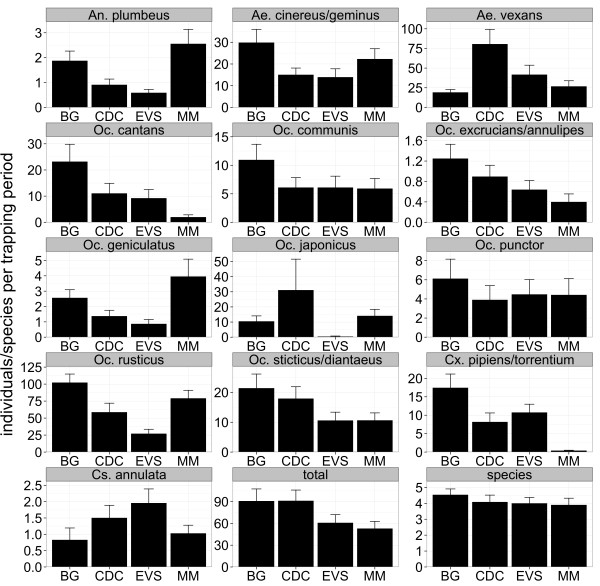
**Number of trapped individuals per species and the total number of individuals among the four trapping devices.** Mean +/-SE number of trapped individuals per trapping period for each species and the total number of individuals and the mean +/-SE number of species among the four trapping devices. Only mosquito species caught with more than 100 individuals are shown and trapping periods were only included if the species was detected with at least one individual in the corresponding trapping period at the sampling location.

**Figure 5:**
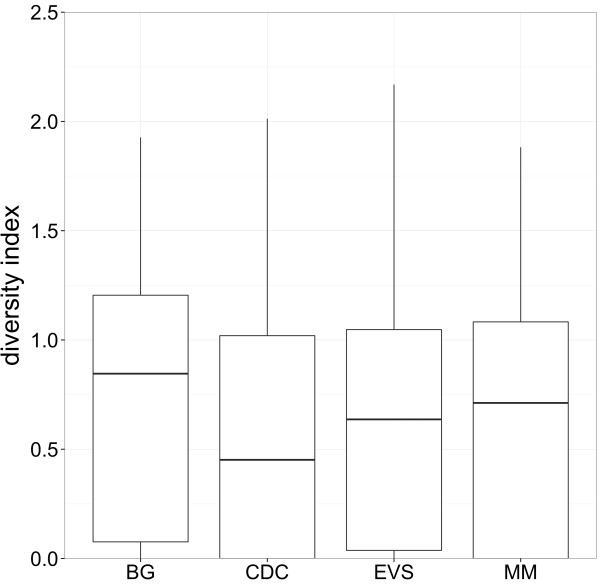
**Simpson’s diversity index among the four trapping devices.** Boxplots of Simpson’s diversity indices per trapping period among the four trapping devices.

During the study 24,094 mosquitoes were caught, belonging to 21 species or morphologically indistinguishable pairs of species (Table 2) and comprising 43% of the established 49 mosquito species in Germany (Table 2, Additional file 1) . All species known to be abundant in Germany and to occur in high density were detected (Table 2) [38]. Most abundant species were *Aedes vexans* (30.0%), *Aedes cinereus*/*geminus* (17.0%), *Culex pipiens*/*torrentium* (12.2%), *Ochlerotatus sticticus*/*diantaeus* (9.9%) and *Ochlerotatus cantans* (9.7%). *Culex hortensis*, *Culex territans*, and *Culiseta morsitans* were only caught with one individual. Undetected species are predominantly classified as less common in Germany (Additional file 1). The BG trap showed the best performance for individuals of the genus *Culex* and the MM trap for the genus *Anopheles* (Figure 2, Table 3). During the entire study the highest number of species was caught with the CDC trap followed by the BG trap, EVS trap, and MM trap, but the total number of species detected was quite similar between the four trapping devices (Figure 3). However, the BG trap caught significantly more species per trapping period compared to CDC trap, EVS trap, and MM trap, while there were no significant differences between the latter three traps (Figure 4, Table 4). This was also supported by slightly higher species diversity indices for the BG trap (Figure 5).

**Table 2:** Number and percentage of trapped individuals for the mosquito species caught with the four different trapping devices

**Species**	**BG**	**%**	**CDC**	**%**	**EVS**	**%**	**MM**	**%**	**Total**	**Occurence in Germany**
*Anopheles maculipennis* s.l.*	0	0.0	18	33.3	18	33.3	18	33.3	54	+++
*Anopheles claviger*	2	3.1	33	50.8	9	13.8	21	32.3	65	++
*Anopheles plumbeus*	105	33.1	51	16.1	33	10.4	128	40.4	317	++
*Aedes cinereus*/*geminus*	1,552	38.0	783	19.2	725	17.7	1,027	25.1	4,087	++/proven
*Aedes rossicus*	6	66.7	3	33.3	0	0.0	0	0.0	9	++
*Aedes vexans*	841	11.6	3,544	49.0	1,837	25.4	1,016	14.0	7,238	++++
*Ochlerotatus cantans*	1,206	51.9	565	24.3	470	20.2	84	3.6	2,325	++
*Ochlerotatus caspius*	1	8.3	10	83.3	1	8.3	0	0.0	12	(+)
*Ochlerotatus communis*	208	39.8	116	22.2	116	22.2	83	15.9	523	+
*Ochlerotatus excrucians*/*annulipes*	50	41.0	35	28.7	25	20.5	12	9.8	122	(+)/++
*Ochlerotatus geniculatus*	144	29.8	77	15.9	49	10.1	214	44.2	484	(+)
*Ochlerotatus japonicus*	84	18.7	249	55.3	4	0.9	113	25.1	450	+
*Ochlerotatus punctor*	141	35.6	90	22.7	103	26.0	62	15.7	396	+
*Ochlerotatus rusticus*	818	38.3	470	22.0	217	10.1	633	29.6	2,138	++
*Ochlerotatus sticticus*/*diantaeus*	857	36.0	718	30.1	424	17.8	384	16.1	2,383	+++/(+)
*Ochlerotatus* spec.	30	68.2	6	13.6	3	6.8	5	11.4	44	
*Culex hortensis*	0	0.0	1	100.0	0	0.0	0	0.0	1	-
*Culex pipiens*/*torrentium*	1,398	47.5	655	22.3	861	29.3	29	1.0	2,943	++++/++++
*Culex territans*	0	0.0	1	100.0	0	0.0	0	0.0	1	++
*Culiseta annulata*	59	16.0	107	29.0	139	37.7	64	17.3	369	++
*Culiseta morsitans*	1	100.0	0	0.0	0	0.0	0	0.0	1	+
*Culiseta* spec.	2	66.7	0	0.0	0	0.0	1	33.3	3	
*Coquillettidia richiardii*	17	21.8	14	17.9	20	25.6	27	34.6	78	+
Unidentified Culicidae	18	35.3	27	52.9	5	9.8	1	2.0	51	
**Total**	**7**,**540**	**31.3**	**7**,**573**	**31.4**	**5**,**059**	**21.0**	**3**,**922**	**16.3**	**24**,**094**	

Number and percentage of trapped individuals for the mosquito species caught with the four different trapping devices. Occurrence in Germany classified after Becker *et al.*[38] (occurrence: ++++ = massive; +++ = abundant; ++ = frequent; + = regularly; (+) = rare; - = not classified; *species complex includes *Anopheles atroparvus*, *An. daciae*, *An. maculipennis*, *An. messeae*).

**Table 3:** Statistical differences between the number of trapped individuals per genera among the four trapping devices

**Response variable**	**Traps**	**Estimate**	**SE**	**DF**	**t**	**p**
*Anopheles*	BG vs. MM	-0.905	0.349	300.7	-2.59	0.010
	CDC vs. MM	-0.950	0.349	300.6	-2.72	0.007
	EVS vs. MM	-1.456	0.349	300.6	-4.17	<0.001
*Aedes*/						
*Ochlerotatus*	BG vs. EVS	23.570	10.264	299.1	2.30	0.022
	BG vs. MM	23.796	10.654	299.5	2.23	0.026
	CDC vs. EVS	32.398	10.260	299.0	3.16	0.002
	CDC vs. MM	32.624	10.650	299.5	3.06	0.002
*Culex*	BG vs. CDC	8.933	2.807	299.2	3.18	0.002
	BG vs. EVS	6.475	2.807	299.2	2.31	0.022
	BG vs. MM	16.962	2.911	300.5	5.83	<0.001
	CDC vs. MM	8.029	2.910	300.5	2.76	0.006
	EVS vs. MM	10.486	2.910	300.5	3.60	0.000
*Culiseta*	BG vs. EVS	-0.963	0.328	298.5	-2.93	0.004

Mean +/-SE differences in least squares means associated with the mixed linear models for the number of individuals per trapping period among the four trapping devices. Only mosquito genera caught with more than 100 individuals are shown and trapping periods were only included if the genus was detected with at least one individual in the corresponding trapping period at the sampling location (only significant differences shown). BG: Biogents Sentinel trap, EVS: Heavy Duty Encephalitis Vector Survey trap, CDC: Centres for Disease Control miniature light trap, MM: Mosquito Magnet Patriot Mosquito trap, Estimate: differences in least squares means, SE: standard error, DF: degrees of freedom, t: t-value, p: p value.

**Table 4:** Statistical differences between the number of trapped individuals per species and the total number of individuals among the four trapping devices

**Response variable**	**Traps**	**Estimate**	**SE**	**DF**	**t**	**p**
*Anopheles plumbeus*	BG vs. CDC	0.641	0.299	301.2	2.14	0.033
	BG vs. EVS	0.858	0.299	301.2	2.87	0.004
	CDC vs. MM	-1.032	0.307	302.1	-3.36	0.001
	EVS vs. MM	-1.249	0.307	302.1	-4.07	<0.001
*Aedes cinereus/geminus*	BG vs. CDC	9.304	2.354	301	3.95	<0.001
	BG vs. EVS	10.002	2.354	301	4.25	<0.001
	BG vs. MM	6.052	2.418	301.3	2.5	0.013
*Aedes vexans*	BG vs. CDC	-32.42	7.296	301.1	-4.44	<0.001
	CDC vs. EVS	20.566	7.293	301.1	2.82	0.005
	CDC vs. MM	28.547	7.49	301.7	3.81	<0.001
*Ochlerotatus cantans*	BG vs. CDC	7.732	2.456	301.1	3.15	0.002
	BG vs. EVS	8.877	2.456	301.1	3.61	<0.001
	BG vs. MM	13.929	2.524	301.4	5.52	<0.001
	CDC vs. MM	6.197	2.523	301.4	2.46	0.015
	EVS vs. MM	5.052	2.523	301.4	2	0.046
*Ochlerotatus communis*	BG vs. CDC	1.11	0.427	301	2.6	0.01
	BG vs. EVS	1.11	0.427	301	2.6	0.01
	BG vs. MM	1.497	0.439	301.2	3.41	0.001
*Ochlerotatus excrucians/annulipes*	BG vs. EVS	0.299	0.138	301.3	2.17	0.031
	BG vs. MM	0.418	0.142	302.6	2.95	0.003
*Ochlerotatus geniculatus*	BG vs. EVS	1.144	0.466	301.1	2.45	0.015
	BG vs. MM	-1.039	0.479	301.6	-2.17	0.031
	CDC vs. MM	-1.845	0.479	301.6	-3.85	<0.001
	EVS vs. MM	-2.183	0.479	301.6	-4.56	<0.001
*Ochlerotatus punctor*	BG vs. MM	0.976	0.334	301.2	2.92	0.004
*Ochlerotatus rusticus*	BG vs. CDC	4.198	1.764	301	2.38	0.018
	BG vs. EVS	7.246	1.764	301	4.11	<0.001
	EVS vs. MM	-5.159	1.812	301.2	-2.85	0.005
*Ochlerotatus sticticus/diantaeus*	BG vs. EVS	5.248	1.373	301	3.82	<0.001
	BG vs. MM	5.675	1.411	301.2	4.02	<0.001
	CDC vs. EVS	3.542	1.373	301	2.58	0.01
	CDC vs. MM	3.969	1.411	301.2	2.81	0.005
*Culex pipiens/torrentium*	BG vs. CDC	8.957	2.797	301.1	3.2	0.002
	BG vs. EVS	6.475	2.797	301.1	2.31	0.021
	BG vs. MM	17.026	2.873	302	5.93	<0.001
	CDC vs. MM	8.069	2.872	301.9	2.81	0.005
	EVS vs. MM	10.55	2.872	301.9	3.67	<0.001
*Culiseta annulata*	BG vs. EVS	-0.975	0.347	300.8	-2.81	0.005
	EVS vs. MM	0.703	0.357	301.4	1.97	0.05
Total	BG vs. EVS	30.106	10.598	301	2.84	0.005
	BG vs. MM	41.781	10.889	301.3	3.84	<0.001
	CDC vs. EVS	30.289	10.595	301	2.86	0.005
	CDC vs. MM	41.965	10.885	301.3	3.86	<0.001
Species	BG vs. CDC	0.446	0.215	301	2.08	0.039
	BG vs. EVS	0.531	0.215	301	2.47	0.014
	BG vs. MM	0.619	0.221	301.1	2.8	0.005

Mean +/-SE differences in least squares means associated with the mixed linear models for the number of trapped individuals per trapping period for each species and the total number of individuals and the mean +/-SE number of species among the four trapping devices. Only mosquito species caught with more than 100 individuals are shown and trapping periods were only included if the species was detected with at least one individual in the corresponding trapping period at the sampling location (only significant differences shown). BG: Biogents Sentinel trap, EVS: Heavy Duty Encephalitis Vector Survey trap, CDC: Centres for Disease Control miniature light trap, MM: Mosquito Magnet Patriot Mosquito trap, Estimate: differences in least squares means, SE: standard error, DF: degrees of freedom, t: t-value, p: p value.

BG and CDC traps caught significantly more mosquitoes per trapping period compared to EVS and MM traps (Figure 4, Table 4). The four trapping devices differed in performance regarding their efficacy to trap individual mosquito species. The BG trap caught significantly more individuals of the species *Oc. cantans*, *Ae. cinereus*/*geminus*, *Oc. communis*, and *Cx. pipiens*/*torrentium* per trapping period. The CDC trap outcompeted the other devices by trapping significantly more *Ae. vexans* individuals per trapping period and the MM trap caught significantly more individuals of *Oc. geniculatus* per trapping period. In contrast, the EVS trap did not outperform for any species. The MM trap caught the smallest number of individuals of the species *Cx. pipiens*/*torrentium* and *Oc. cantans* per trapping period. Additionally, the CDC trap with light outcompeted the EVS trap without light for *Aedes vexans* and *Ochlerotatus sticticus*/*diantaeus*.

**Figure 6:**
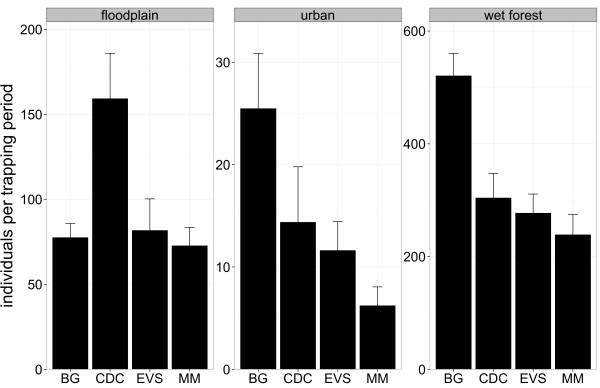
**Number of individuals per aggregated biotope among the four trapping devices.** Mean +/-SE number of trapped individuals per trapping period among the four trapping devices and the three aggregated biotopes.

**Figure 7:**
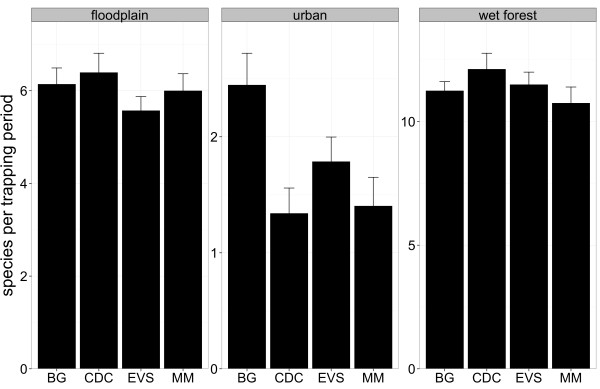
**Number of species among the four trapping devices and aggregated biotopes.** Mean +/-SE number of trapped species per trapping period among the four trapping devices and the three aggregated biotopes.

The four traps showed differences in their suitability for the three aggregated biotopes investigated. The BG trap caught significantly more individuals per trapping period in the urban environment as well as in wet forest (Figure 6, Table 5), while the CDC trap caught most individuals per trapping period in the floodplain. Moreover, the BG trap was most efficient for the trapping of the variety of mosquito species per trapping period in an urban environment (Figure 7, Table 6).

**Table 5:** Statistical differences between the number of trapped individuals among the four trapping devices and aggregated biotopes

**Response variable**	**Biotope**	**Traps**	**Estimate**	**SE**	**DF**	**t**	**p**
Total	Floodplain	BG vs. CDC	-81.304	22.617	98.2	-3.59	0.001
	Floodplain	CDC vs. EVS	77.536	22.597	98.1	3.43	0.001
	Floodplain	CDC vs. MM	82.400	23.742	99.2	3.47	0.001
	Urban	BG vs. CDC	11.106	5.247	168.1	2.12	0.036
	Urban	BG vs. EVS	13.851	5.247	168.1	2.64	0.009
	Urban	BG vs. MM	20.203	5.430	169.1	3.72	<0.001
	Wet forest	BG vs. CDC	216.625	38.277	27.0	5.66	<0.001
	Wet forest	BG vs. EVS	243.375	38.277	27.0	6.36	<0.001
	Wet forest	BG vs. MM	281.750	38.277	27.0	7.36	<0.001

Mean +/-SE differences in least squares means associated with the mixed linear models for the number of trapped individuals per trapping period among the four trapping devices and the three aggregated biotopes (only significant differences shown). BG: Biogents Sentinel trap, EVS: Heavy Duty Encephalitis Vector Survey trap, CDC: Centres for Disease Control miniature light trap, MM: Mosquito Magnet Patriot Mosquito trap, Estimate: differences in least squares means, SE: standard error, DF: degrees of freedom, t: t-value, p: p value.

**Table 6:** Statistical differences between the number of trapped species among the four trapping devices and aggregated biotopes

**Response variable**	**Biotope**	**Traps**	**Estimate**	**SE**	**DF**	**t**	**p**
Species	Urban	BG vs. CDC	1.106	0.249	168.0	4.44	<0.001
	Urban	BG vs. EVS	0.660	0.249	168.0	2.65	0.009
	Urban	BG vs. MM	0.981	0.258	168.5	3.80	<0.001

Mean +/-SE differences in least squares means associated with the mixed linear models for the number of trapped species per trapping period among the four trapping devices and the three aggregated biotopes (only significant differences shown). BG: Biogents Sentinel trap, EVS: Heavy Duty Encephalitis Vector Survey trap, CDC: Centres for Disease Control miniature light trap, MM: Mosquito Magnet Patriot Mosquito trap, Estimate: differences in least squares means, SE: standard error, DF: degrees of freedom, t: t-value, p: p value.

## Discussion

Several commercial trapping devices for mosquitoes are available, which are used for nuisance reduction, mosquito monitoring, or surveillance. This study compared the performance of four aspiration traps in Germany (Biogents Sentinel trap (BG trap), Heavy Duty Encephalitis Vector Survey trap (EVS trap), Centres for Disease Control miniature light trap (CDC trap), and Mosquito Magnet Patriot Mosquito trap (MM trap)), which are commonly used in Central Europe. During the study period we found all mosquito species, which are known to be abundant in Germany. The four traps detected a similar number of species. However, the BG trap was found to trap the largest diversity of mosquito species per trapping period, while there were no differences between the other three traps. Additionally, the BG and CDC traps caught more mosquito individuals per trapping period than the EVS and MM traps. This matches a trap comparison from the Netherlands, which found the CDC trap catching more individuals than the MM trap [20]. In the same study, the CDC trap was found to catch more species than the MM trap, a result that is not supported by our study. Our results are also in contrast to a trap comparison from Great Britain, which identified the MM trap to catch more species and individuals than the CDC trap [19].

Contrary to a Spanish study [22], which did not find differences between CDC and BG traps in collecting *Cx. pipiens*, our study indicated that the BG trap caught significantly more *Cx. pipiens*/*torrentium* per trapping period compared to the other three trapping devices. Our findings are in agreement with results of Reusken *et al.*[20], who showed a very low trapping efficacy of the MM trap for *Cx. pipiens*/*torrentium*, while Drago *et al.*[10] did not find significant differences between BG and CDC traps for these species. Furthermore, the BG trap outperformed the other three traps for the three floodwater species *Oc. cantans*, *Ae. cinereus*/*geminus* and *Oc. communis*. A high trapping efficacy of the BG trap for members of the genera *Aedes* and *Ochlerotatus* was supported by several studies [reviewed by 10]. The MM trap also had the lowest performance for *Oc. cantans*.

The CDC and MM traps performed better than the other three traps for one particular species *each*. The CDC trap caught the highest number of *Ae. vexans* per trapping period, which matches the results of a study from the U.S. [39], in which the CDC trap performed better than the BG trap. In contrast, another U.S. study did not find clear differences between the BG and CDC trap [14]. Our study showed that the MM trap caught the highest number of *Oc. geniculatus*, which is in agreement with a study from the U.K., in which the MM trap caught more individuals of this species compared to the CDC trap [19].

The four trapping devices used in this study differ in constructions and mechanisms to lure and trap mosquitoes. Except for the addition of a dry ice bucket to the CDC trap, we used the traps according to the manufacturers’ instructions and did not interfere with recommended trap configurations (e.g. with lure vs. without lure or different heights). However, changes of configurations might result in a different performance of the various traps.

Although the CO_2_-effusion rate from dry ice (CDC and EVS traps) is probably more temperature-dependent than from gas cylinders (BG trap), this probably does not have a strong impact on the trapping efficacy. The same probably applies to the amount of CO_2_ and the type of CO_2_ dispersal from small holes on the EVS dry ice bucket (EVS and CDC traps) or tubes (BG and MM traps) [13], which should not cause profound differences between the traps. However, it is surprising that the CDC trap revealed significantly higher trapping efficacy for the species *Ae. vexans* and *Oc. sticticus*/*diantaeus* compared to the rather similar EVS trap. The trap cover of the CDC trap (33 cm in diameter) has a positive impact on the diffusion range of the CO_2_[40], which may result in significant trapping differences for this very abundant species. Another explanation could be the secondary attractant of light, which is only used with the CDC trap. However, the study by Becker *et al.*[41] did not reveal a significant impact of light on the trapping efficacy of the CDC trap for *Aedes*.

The BG and MM traps use different chemical lures additional to carbon dioxide to increase their trapping efficacy by imitating the olfactory cues of potential hosts (e.g. octenol for ruminant breaths or lactic acid as component of sweat) [42]. Such lures can have significant influences on the trapping efficacy for particular mosquito species, but do not necessarily cause differences [21,22,42]. Only for *An. plumbeus* there was a a significantly better performance for the two lure-containing traps (BG and MM traps) compared to those without lure (CDC and EVS traps). However, with the exception of *Oc. geniculatus*, the BG trap performed similar or even better compared to the MM trap. Although the BG Lure used for the BG traps might explain the better performance for some of the species, it probably does not explain the differences for all of them, as there were no significant differences for the trapping of *Cx. pipiens* with and without BG Lure in a previous German study [21].

We conducted our trap comparison at ten sampling locations distributed in northern and southern Germany, which were analysed as aggregated biotopes for floodplain, urban and wet forest, respectively. According to its trapping performance for *Culex* species, we found the BG trap to be superior in an urban environment. The CDC trap was the most efficient trapping device for *Aedes vexans* and therefore should be the first choice for the floodplain environment and the BG trap showed an outstanding performance for some of the snow-melt mosquito species and therefore trapped most mosquito individuals in the wet forest.

## Conclusion

This study compared four adult mosquito traps (BG trap, EVS trap, CDC trap, and MM trap) under different environmental conditions in Germany with a total of 323 24-hour sampling periods (83 × BG trap, 83 × CDC trap, 83 × EVS trap, 74 × MM trap) and the analysis of more than 24,000 mosquitoes from 21 species most common in Central Europe. The BG trap showed the best performance regarding the number of mosquitoes and the number of mosquito species per trapping period and outperformed the other three traps for the genus *Culex* and for four species (*Oc. cantans*, *Ae. cinereus*/*geminus*, *Oc. communis* and *Cx. pipiens*/*torrentium*). The CDC trap was the most efficient trap for *Ae. vexans* and the MM trap for the genus *Anopheles* and the species *Oc. geniculatus*. The EVS trap did not show advantages for any species or genus compared to the other three traps. Additionally, the MM trap had a very low efficacy for *Cx. pipiens*/*torrentium* and *Oc. cantans*. According to its efficacy for the number of mosquitoes and the range of species at various environments, the BG trap is recommended as the general monitoring trapping device for common mosquito species in Central Europe, while the CDC trap is the best choice to trap large numbers of mosquitoes particularly in floodplain biotopes.

## Competing interests

Over the last three years, the company Biogents, which is the producer of the BG mosquito traps included in this study, was a research partner of the authors NB, KH, EK, AK, RL, WPP and ET in a surveillance study for invasive mosquitoes in Germany. Biogents had no role in study design, data collection and analysis, preparation of the manuscript or decision on publication.

## Authors’ contributions

Designed the study: AK, RL, WPP, EK. Performed the data collection: JB, RG, KH, AK, RL, NS, SS, NB, ET, WPP. Analysed the data: RL. Wrote the paper: RL. Contributed to the manuscript drafting: AK, ET, WPP, RG, EK, NS, SS. All authors read and approved the final version of the manuscript.

## Supplementary Material

Additional file 1 **Mosquito species not caught with the four different trapping devices.** Description of data: Mosquito species not caught with the four different trapping devices. Occurrence in Germany classified after Becker *et al.*[38] (occurrence: ++ = frequent; + = regularly; (+) = rare; - = not classified; * species is not established; [] = not counted in the species lists). Click here for file
