# Supplementary material for: Field evaluation of four widely used mosquito traps in Central Europe
Source: Parasit Vectors. 2014 Jun 12;7:268. doi: 10.1186/1756-3305-7-268 (PMC4064298; doi:10.1186/1756-3305-7-268)
Supplement: Additional file 1 — Mosquito species not caught with the four different trapping devices. Description of data: Mosquito species not caught with the four different trapping devices. Occurrence in Germany classified after Becker et al.[38] (occurrence: ++ = frequent; + = regularly; (+) = rare; - = not classified; * species is not established; [] = not counted in the species lists). [file 1756-3305-7-268-S1.docx]

## Table - Mosquito species not caught with the four different trapping devices

Mosquito species not caught with the four different trapping devices. Occurrence in Germany classified after Becker et al. [36] (occurrence: ++ = frequent; + = regularly; (+) = rare; - = not classified; * species is not established; [ ] = not counted in the species lists).

| **Species** | **Occurence in Germany** |
| --- | --- |
| *An. algeriensis* | - |
| *Ae. albopictus** | [(+)] |
| *Oc. cataphylla* | (+) |
| *Oc. cyprius* | - |
| *Oc. dorsalis* | (+) |
| *Oc. detritus* | (+) |
| *Oc. flavescens* | (+) |
| *Oc. intrudens* | - |
| *Oc. leucomelas* | + |
| *Oc. nigrinus* | (+) |
| *Oc. refiki* | - |
| *Oc. riparius* | - |
| *Oc. pullatus* | (+) |
| *Cx. martinii* | - |
| *Cx. modestus* | ++ |
| *Cs. alascaensis* | (+) |
| *Cs. fumipennis* | - |
| *Cs. glaphyroptera* | (+) |
| *Cs. longiareolata* | (+) |
| *Cs. subochrea* | (+) |
| *Cs. ochroptera* | + |
| *Ur. unguiculata* | (+) |
